# Multidrug Efflux Pumps at the Crossroad between Antibiotic Resistance and Bacterial Virulence

**DOI:** 10.3389/fmicb.2016.01483

**Published:** 2016-09-21

**Authors:** Manuel Alcalde-Rico, Sara Hernando-Amado, Paula Blanco, José L. Martínez

**Affiliations:** Departamento de Biotecnología Microbiana, Centro Nacional de Biotecnología, Consejo Superior de Investigaciones CientíficasMadrid, Spain

**Keywords:** multidrug efflux pumps, quorum sensing, antibiotic resistance mechanisms, virulence, global regulation

## Abstract

Multidrug efflux pumps can be involved in bacterial resistance to antibiotics at different levels. Some efflux pumps are constitutively expressed at low levels and contribute to intrinsic resistance. In addition, their overexpression may allow higher levels of resistance. This overexpression can be transient, in the presence of an effector (phenotypic resistance), or constitutive when mutants in the regulatory elements of the expression of efflux pumps are selected (acquired resistance). Efflux pumps are present in all cells, from human to bacteria and are highly conserved, which indicates that they are ancient elements in the evolution of different organisms. Consequently, it has been suggested that, besides antibiotic resistance, bacterial multidrug efflux pumps would likely contribute to other relevant processes of the microbial physiology. In the current article, we discuss some specific examples of the role that efflux pumps may have in the bacterial virulence of animals’ and plants’ pathogens, including the processes of intercellular communication. Based in these evidences, we propose that efflux pumps are at the crossroad between resistance and virulence of bacterial pathogens. Consequently, the comprehensive study of multidrug efflux pumps requires addressing these functions, which are of relevance for the bacterial–host interactions during infection.

## Introduction

Multidrug resistance (MDR) efflux pumps are relevant elements belonging to the microbial repertoire that bacteria harbor for resisting the action of antimicrobial drugs (Piddock, 2006a; Vila and Martínez, 2008; Li et al., 2015; Jang, 2016). Indeed, several works have shown that these elements are involved in resistance of *in vitro* selected mutants as well as in the reduced susceptibility to antimicrobials of clinical isolates of different bacterial pathogens. The expression of efflux pumps is usually down regulated; only some of them present a substantial level of expression under regular growing conditions in the laboratory (Grkovic et al., 2001, 2002). However, constitutive high-level expression of these elements can be achieved by means of mutations in the elements regulating their expression. Transient high-level expression of efflux pumps can also be triggered in the presence of their effectors or under some specific growing conditions. In agreement with this situation, efflux pumps contribute to antibiotic resistance at three different levels: they can be involved in intrinsic resistance when presenting a basal level of expression under any condition. They can contribute to acquired resistance when mutants achieving high-level of expression of the efflux pumps are selected. Finally, they can contribute to transient, non-inheritable, phenotypic resistance when bacteria are growing in the presence of an effector of the efflux pump or under growing conditions that trigger their overexpression. As reviewed in Hernando-Amado et al. (2016), efflux pumps are grouped in five structural families, namely the resistance-nodulation-division (RND), the small multidrug resistance (SMR), the multi antimicrobial extrusion (MATE), the major facilitator superfamily (MFS), and the ATP-binding cassette (ABC) superfamilies. Whereas some efflux pumps can work independently of any other protein, mainly in the case of Gram-positive organisms, in the case of Gram-negative organisms, they form tripartite complexes capable to traverse both bacterial membranes. These complexes include the inner-membrane efflux pump, a membrane fusion protein and an outer membrane protein.

When compared with other classical resistance genes, MDR efflux pumps present some specific features that support they should have other roles in the bacterial physiology besides their well-known involvement in antibiotic resistance. First, MDR efflux pumps are ubiquitous; they are present in all living cells, from humans to bacteria (Alonso et al., 1999; Alonso and Martinez, 2001; Gould et al., 2004; Sanchez et al., 2004). Second, the genes encoding them belong to the bacterial core genome in the sense that all (or most) members of a given species harbor the same efflux pumps (Alonso et al., 1999). Third, they are redundant; a single bacterial cell usually contains more than 10 different efflux pumps (Crossman et al., 2008). Fourth, they are rather unspecific; each efflux pump is able to extrude a variety of different substrates, including synthetic antibiotics as quinolones (Hernandez et al., 2011; Redgrave et al., 2014). Fifth, as above mentioned the expression of efflux pumps is tightly regulated; this regulation includes local regulators usually encoded upstream the structural genes of the operon encoding the efflux pump, as well as global regulators (Randall and Woodward, 2002; Luong et al., 2003; Nikaido et al., 2008; De Majumdar et al., 2013), frequently controlling the expression of a set of genes involved in the adaptation to a given ecosystem, as is the infected host. Sixth, at least in occasions, antibiotics are not good effectors of the expression of efflux pumps, whereas host-produced compounds as bile salts or plant-produced signals may induce the expression of MDR pumps (Rosenberg et al., 2003; Prouty et al., 2004; García-León et al., 2014). Altogether, these characteristics support that MDR efflux pumps are ancient elements (present in all organisms), important for the bacterial physiology (all members of a given species present the same, conserved efflux pumps), likely displaying different functions besides antibiotic resistance (a single microorganism contains a large number of different efflux pumps, with overlapping substrate ranges, including synthetic antibiotics not present in nature) and frequently integrated in complex response networks (they form part of global regulons and their expression is triggered by host produced compounds). In the present article we discuss some examples of the potential functions, besides antibiotic resistance, of MDR efflux pumps with a particular focus on the role that they may have in bacterial–host interactions in animals (humans) and plants as well as in intercellular signaling (Piddock, 2006b; Martinez et al., 2009; Alvarez-Ortega et al., 2013).

## Efflux Pumps and Cell-To-Cell Communication

The capability to sense the environment and the organisms that are living in the same niche is critical to allow the microorganisms for choosing the best strategy to survive and colonize such niche. Along evolution, a battery of different mechanisms to sense the continuously changing environment has been selected in different microbial species. One of these mechanisms consists on the cell-to-cell communication systems. These inter-cellular signaling systems are based on the production of one or more low-molecular weight compounds which are sensed by molecular receptors of other cells, promoting a specific response in the target organism. In the bacterial world, this phenomenon is known as quorum sensing (QS) because it was initially described as a mechanism to sense the density of the bacterial population belonging to the same species present in a given habitat. The QS system allows the establishment of a cooperative genetic program of the whole population that increases the microbial efficacy for colonizing a given environment, including the infected host (Williams et al., 2007). The QS signal molecules (QSSMs) are constitutively produced at low quantities by all cells in the population. Their release outside the cells allows a progressive accumulation of QS signals in the intercellular space. The consequence is an increasingly production and accumulation of QSSMs while the population size increases, activating the QS response when the concentration of the signal reaches a threshold level. Further, these molecules (known as autoinducers) are able to induce their own production when they bind to their cognate transcriptional regulators, which produce a feed-forward regulation circuit. The regulator-QSSM complex is the main responsible of triggering the QS response by increasing the expression of a large number of genes, including those encoding the autoinducer synthase enzymes, which further increases the QSSM production. This response coordinates a number of physiological changes at a population-scale level, which allows among other issues improving bacterial competition for nutrients with other species, forming morphological-resistance structures to overcome environmental threats or triggering the expression of virulence factors (Swift and Downie, 2001; Williams, 2007). Further, it has been shown that several QSSMs can be sensed by other species promoting responses mediated by inter-species communication processes (Williams et al., 2007; Shimada et al., 2013; Lee and Zhang, 2015), including eukaryotic cells (interkingdom signaling; Martinez, 2014). As above stated these QSSMs have to cross the cells membranes for their extra-cellular accumulation, and it has been reported that some MDR efflux pumps might be involved in their transport outside the cell. In this section, we will discuss the role of MDR efflux pumps in the modulation of intercellular signaling and how the acquisition of antibiotic resistance in mutants overexpressing efflux pumps may challenge bacterial virulence through alterations in the diffusion of QS signals.

**FIGURE 1:**
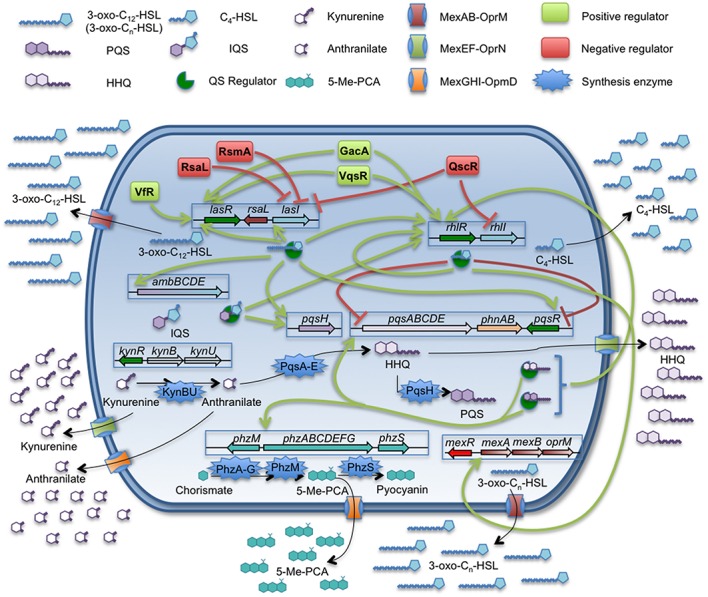
**Quorum sensing network and related efflux pumps in *P. aeruginosa*.** The QS signals produced by *P. aeruginosa* are: 3-oxo-C_12_-HSL, C_4_-HSL, PQS/HHQ, and the recently discovered IQS. The figure sums up the complexity of the QS regulation network and the implications of MexAB-OprM, MexEF-OprN, and MexGHI-OpmD efflux pumps on the extrusion of QSSMs, their precursors or molecules which expression is QS-regulated. Despite of C_4_-HSL is able to cross the cell envelopes by free diffusion, the expression of *mexAB-oprM* is triggered in presence of this autoinducer. Further, this efflux pump is able to extrude 3-oxo-C_12_-HSL and others related 3-oxo-C_n_-HSL. The MexEF-OprN system is able to extrude HHQ and kynurenine, a precursor of 4-alkyl-quinolones, having an impact on the QS response. In the case of MexGHI-OpmD, its role has been recently linked to the extrusion of 5-Me-PCA, a precursor of the phenazine pyocyanin, whose production is induced upon the QS response. However, it has been proposed that this system is able to efflux anthranilic acid, another AQs intermediate, which is toxic for the cell at high concentrations. *P. aeruginosa* is a good example for the potential role of efflux systems in modulating the cell-to-cell communication networks.

*Pseudomonas aeruginosa* is one of the most important opportunistic pathogens causing infections at hospitals as well as in cystic fibrosis patients (Buhl et al., 2015; Kaye and Pogue, 2015; Oliver et al., 2015; Talwalkar and Murray, 2016). This pathogen is able to produce different virulence factors, many of them being regulated by a hierarchically organized QS signaling system (**Figure 1**), which consists in three different and interconnected regulatory networks each one respectively governed by the transcriptional regulators LasR, RhlR, and PqsR (Lee and Zhang, 2015). These three QS regulators recognize respectively one of different QSSMs produced by *P. aeruginosa*: *N*-(3-oxododecanoyl)-L-homoserine lactone (3-oxo-C12-HSL), *N*-butanoyl-L-homoserine lactone (C4-HSL), and *Pseudomonas* quinolone signal (PQS). The presence of an additional regulatory QS network has been recently described (Lee et al., 2013; Lee and Zhang, 2015). Despite being tightly regulated by the *las* system in standard conditions of growth, this new system is able to trigger the PQS and C4-HSL production in absence of the *las* system or under phosphate stress conditions. However, the mechanisms responsible of the regulation mediated by this QS system are not fully understood and consequently will not be discussed along the present review.

Some works have shown that the expression of different efflux pumps encoded in the *P. aeruginosa* chromosome may have an impact in the QS-regulation networks of this microorganism. For instance, the RND efflux pump MexAB-OprM is highly integrated within the *las* and *rhl* QS regulons, since it has been shown that its expression can be induced by C4-HSL (Maseda et al., 2004; Sawada et al., 2004). In addition, this efflux pump is able to extrude the 3-oxo-C12-HSL QS signal (Evans et al., 1998; Pearson et al., 1999; Minagawa et al., 2012). In such a way, the constitutive high-level expression of MexAB-OprM entails the extrusion of 3-oxo-C12-HSL, increasing the concentration of this QS signal around the cell, but reducing its intracellular accumulation. Indeed, antibiotic resistant mutants overexpressing MexAB-OprM present defects in the production of several virulence factors and are impaired in their QS response (Evans et al., 1998).

It is important to highlight that the 3-oxo-C12-HSL autoinducer is not just an intra-specific signal compound but may also entail inter-kingdom signaling. For instance, it has been reported that this QS signal may inhibit the filamentous differentiation, which is linked to a virulent state of *Candida albicans*, a fungal pathogen commonly found in patients with *P. aeruginosa* infections (Hogan et al., 2004). Conversely, it has been also shown that the *C. albicans* QS compound farnesol in turn can inhibit the production of QSSMs and the production of virulence factors by *P. aeruginosa* (Méar et al., 2013), evidencing the relevance of these cell-to-cell communication “weapons” in the establishment of a competitive interaction between these two opportunistic pathogens. In addition to its role in inter-microbial interactions, a role as activator of the human immune system has been attributed to 3-oxo-C12-HSL. This autoinducer signal can act as a chemoattractant for polymorphonuclear neutrophils and is able to induce the expression of adhesion proteins and immunoglobulin receptors implicated in the recognition and localization of microbial infections (Smith et al., 2001; Zimmermann et al., 2006; Wagner et al., 2007). On the other hand, it has been shown that 3-oxo-C12-HSL can induce the apoptosis of neutrophils and macrophages (Tateda et al., 2003). The fact that 3-oxo-C12-HSL has been detected directly in the sputum of cystic fibrosis patients with *P. aeruginosa* infections indicates that this QS signal might be involved in *in vivo* interkingdom signaling (Erickson et al., 2002; Middleton et al., 2002).

*Pseudomonas aeruginosa* MDR strains overexpressing MexAB-OprM produce lower amounts of virulence factors as well as of 3-oxo-C12-HSL (Evans et al., 1998). In addition, *mexAB-OprM* defective mutants are avirulent in a mouse model of infection as well as in MDCK cells (Hirakata et al., 2002). These results support the notion that this efflux system is directly involved in the appropriate and coordinate *las* response needed for a successful host infection. Further, it has been proposed that this system is used by *P. aeruginosa* to optimize indirectly the specific binding of the LasR regulator to 3-oxo-C12-HSL by extruding other 3-oxo-Cn-HSL, which can affect the *las* network activation because they are able of competing with 3-oxo-C12-HSL for the same binding site in LasR (Minagawa et al., 2012). All these intra- and interspecific responses mediated by 3-oxo-C12-HSL suggest that MexAB-OprM is an important element modulating cell-to-cell communication and host–pathogen interactions. As a consequence, the acquisition of antimicrobial resistance through the overexpression of this system could have an impact, at several levels, on the virulence of *P. aeruginosa*.

Besides MexAB-OprM, other *P. aeruginosa* efflux pumps might be involved in the regulation of the expression of QS-dependent virulence factors. One of them is MexEF-OprN, an efflux pump able to extrude both the QS signal HHQ (Lamarche and Deziel, 2011) and its precursor, kynurenine (Olivares et al., 2012), making it an important element in the QS response (Köhler et al., 2001). As we have mentioned above, the virulence and pathogenicity of *P. aeruginosa* is partially controlled by the PQS communication system. Therefore, it is not surprising that, as it happens in the case of MexAB-OprM, mutants overexpressing MexEF-OprN are affected in the production of QS-regulated virulence factors and, in consequence, they are impaired in the host infection process (Olivares et al., 2012). It is worth mentioning that the extrusion of kynurenine by this system could have an additional role in *P. aeruginosa*–host interaction. It has been shown that the expression of the MexEF-OprN system can be induced upon contact with human airway epithelial cells (Frisk et al., 2004). On the other hand, it has been suggested that *P. aeruginosa* production of kynurenine may have a role in the bacterial resistance to the toxic reactive oxygen species (ROS) produced by neutrophils, a key cell component in the innate immune system and in the inflammatory response in lung infections (Genestet et al., 2014). Based on this situation, it is possible that the increased expression of MexEF-OprN upon contact with the lung epithelial cells may allow *P. aeruginosa* to secrete high levels of kynurenine, thus promoting resistance against the neutrophil ROS production. This possibility, which has not been explored yet, would provide MexEF-OprN a new role in the infected lungs beyond the modulation of the QS response.

Another *P. aeruginosa* efflux pump with potential relevance in the virulence of this pathogen is MexGHI-OprD (Aendekerk et al., 2005; Dietrich et al., 2006). It has been shown that this efflux pump is able of extruding 5-methylphenazine-1-carboxylate (5-Me-PCA), a precursor of the phenazine pyocyanin, and anthranilate, the immediate precursor of PQS (Aendekerk et al., 2005). Further, the *mexGHI-oprD* expression could be induced by 5-Me-PCA and, in consequence, is under the transcriptional control of the QS response. It is worth mentioning that the phenazine extruded by this efflux pump is required for biofilm development by *P. aeruginosa* (Sakhtah et al., 2016). If we take into consideration that biofilms are the regular way of growing of *P. aeruginosa* in the lungs of chronically infected patients (Martinez-Solano et al., 2008; Wagner and Iglewski, 2008), this indicates that MexGHI might have a relevant role in the adaptation of *P. aeruginosa* for colonizing that habitat.

Another relevant pathogen whose virulence has been associated to the extrusion of QS signals by efflux pumps is *Burkholderia pseudomallei*, the causal agent of melioidosis. Different works have suggested that the virulence of this pathogen is modulated by the stationary phase sigma factor RpoS in addition to the regulatory activity of a QS system based on both acyl-homoserine lactones (AHLs) and 2-alkyl-4-quinolones (Ulrich et al., 2004; Wongtrakoongate et al., 2012; Butt et al., 2016). *B. pseudomallei* produces up to six different homoserine lactones: *N*-octanoyl-homoserine lactone (C8-HSL), *N*-decanoyl-homoserine lactone (C10-HSL), *N*-(3-hydroxy)-octanoyl-homoserine lactone (3-OH-C8-HSL), *N*-(3-hydroxy)-decanoyl-homo-serine lactone (3-OH-C10-HSL), *N*-(3-oxo)-decanoyl-homoserine lactone (3-oxo-C10-HSL), and *N*-(3-oxo)-tetradecanoyl-homoserine lactone (3-oxo-C14-HSL). It has been reported that in the clinical strain KHW, the BpeAB-OprB efflux pump is strictly needed for the synthesis and full extrusion of these six AHLs and it has been suggested that another efflux pump, AmrAB-OprA could also be involved in the extrusion of 3-oxo-C10-HSL (Chan et al., 2007). In agreement with a potential role of these efflux pumps in QS communication, it has been shown that *bpeAB-oprB* expression is induced in presence of exogenous C8-HSL and C10-HSL (Chan and Chua, 2005). Moreover, a *bpeAB-oprB* defective mutant is affected in the expression of QS-dependent virulence factors as well as in biofilm formation. In addition, the mutant presents impaired invasiveness and cytotoxicity in both human macrophages and lung epithelial cells. Even though the role of BpeAB-OprB in virulence might be strain-dependent (Mima and Schweizer, 2010), all of the aforementioned results support the hypothesis that some efflux systems could be involved in the modulation of *B. pseudomallei* virulence and in the host–pathogen interactions through the extrusion of QS communication signals or by responding to their presence.

Other signaling networks involved in intra- and inter-specific communication are based in the use of indole and its derived compounds as signal molecules (Bommarius et al., 2013; Shimada et al., 2013; Lee J.H. et al., 2015). Indole is synthesized by plants and by many different bacteria and has different roles depending on the target species. Even though it was proposed that the efflux pump AcrEF could be implicated in indole export in *Escherichia coli* (Kawamura-Sato et al., 1999), other works have shown that indole is able to diffuse easily across the bacterial envelope (Gaede et al., 2005; Pinero-Fernandez et al., 2011) so that a clear role of efflux pumps in indole trafficking remains controversial. However, a crosstalk between virulence and efflux pumps-linked antibiotic resistance mediated through indole signaling is possible. Indeed, indole may affect the expression of efflux pumps, the resistance to antibiotics and the behavior of *Salmonella enterica* serovar Typhimurium when growing inside the host (Nikaido et al., 2012). This bacterial pathogen does not produce indole. However, it usually lives together with indole-producing species in the host and is able to respond to this signal (Nikaido et al., 2011). It is proposed that indole blocks the activity of the RamR transcriptional regulator through the interaction with its C-terminal domain, leading to the overexpression of RamA activator (Nikaido et al., 2011, 2012). This regulatory protein is able to induce the expression of *acrAB*, *acrEF*, and *tolC*, in addition of repressing the expression of some virulence determinants (Bailey et al., 2010; Nikaido et al., 2012). This entails the development of a low-virulence phenotype, which presents, however, increased resistance to antibiotics as well as to host-produced toxic compounds as bile salts and fatty acids, a phenotype closer to a commensal behavior than to an infective one. This is another example of the relationship between the communication signals, with inter-specific function in this case and the expression of efflux pumps, a situation with clear implications in the virulence potential of bacterial pathogens. Indeed, the role of AcrAB-TolC in the virulence of *Salmonella* goes beyond the acquired phenotype upon induction by indole, since, as described below, it has been shown that *acrAB-tolC* deficient mutants have reduced invasiveness in animal models (Buckley et al., 2006; Webber et al., 2009).

## Multidrug Efflux Pumps and their Role in the Virulence of Human Pathogens

During the course of an infection, a bacterial pathogen has to be capable of surviving from the anti-infective defense mechanisms of the host. These mechanisms include, among others, the production of a diverse set of antimicrobial compounds as fatty acids, peptides or even detergents as bile salts, which function is in food uptake, but also present antimicrobial activity (Fernando and Kumar, 2013). In addition of extruding antibiotics regularly used for treating bacterial infections, different MDR efflux pumps have the ability to extrude a wide variety of compounds, including those antimicrobials produced by the host as well as QS signals involved in the regulation of the expression of virulence determinants (see above). Consequently, these efflux pumps are relevant payers on both antibiotic resistance and virulence of bacterial populations (Piddock, 2006b; Martinez et al., 2009; Alvarez-Ortega et al., 2013).

In the case of enteric bacteria, it has been shown that the efflux of several host-derived antimicrobial compounds, such as bile salts, allows the colonization and promotes the bacterial adaptation to the animal intestinal tract. The best-studied system able to confer resistance to bile salts is the *E. coli* RND efflux pump AcrAB-TolC, which is also a major contributor to intrinsic resistance to antibiotics in this organism (Thanassi et al., 1997). Similar roles have been reported for different AcrAB homologs from other Enterobacteriaceae species, such as *S. enterica* serovar Typhimurium. In this case, it has been described that mutants lacking *acrB* and *tolC* are less proficient for adhering, invading and surviving in mouse monocyte macrophages. The same study reported that an *acrB* mutant was able to colonize chicks. However, it was unable to survive gastrointestinally, which suggests that AcrB is not particularly relevant for the early steps of the gut colonization but it is needed for gastrointestinal persistence. On the other hand, the *tolC* mutant was able to colonize and persist in the chicken intestinal tract, but with a much lower efficiency than the wild-type strain, which could be due to the bile hypersensitivity displayed by this mutant (Buckley et al., 2006). A more recent study, using total genome transcriptional analysis, showed that the inactivation of *acrA*, *acrB*, and *tolC* rendered changes in the level of expression of several genes involved in bacterial pathogenicity, further supporting a crosstalk between resistance and virulence (Webber et al., 2009). For instance, the disruption of *acrB* or *tolC* led to a general repression of the SPI-1 pathogenicity island, which promotes the invasion of non-phagocytic intestinal epithelial cells, as well as bacterial survival and persistence within the host, while inactivation of *acrA* is associated with a repression of SPI-2, which promotes the survival and multiplication in phagocytic cells (Dieye et al., 2009). Therefore, inactivation of *acrB* led to the inability to grow anaerobically, which would negatively impact in the capacity to survive in the host gut; as well as a reduced bacterial motility (Webber et al., 2009), which is also an important factor in the *S. enterica* serovar Typhimurium pathogenicity (Khoramian-Falsafi et al., 1990). As above stated, RND efflux systems are tripartite protein complexes formed by an inner membrane protein (the pump itself), an outer membrane protein and a linker, membrane fusion protein (Yamaguchi et al., 2015; Daury et al., 2016). The inner membrane and the membrane fusion proteins are always encoded in the same operon and are supposed to be specifically associated in each efflux pump, while the outer membrane protein can frequently form part of different efflux pumps. Consequently, while a differential response to the inactivation of the outer membrane protein of the complex (TolC in this case) as compared with the other members of the complex is conceivable, the observed differences between the *acrA* and *acrB* are more difficult to explain and suggests that a certain degree of trans-complementation between the components of different efflux pumps may happen. Although some information on this possibility has been published (Smith and Blair, 2014), this is a topic that remains to be studied in detail, although it merits to be addresses if we wish to understand in full the role that efflux pumps may play in the bacterial physiology besides antibiotic resistance.

An homologous of AcrAB is also present in the respiratory tract pathogen *Moraxella catarrhalis*. In addition of contributing to antibiotic resistance, this efflux pump is also involved in the efficient invasion of nasopharyngeal epithelial cells, since mutants lacking *acrA*, *acrB*, or *tolC* present decreased invasion levels as compared to the wild-type strain (Spaniol et al., 2015). This study also showed that exposure to cold shock (26°C) led to an increase in the expression of the efflux pump genes (Spaniol et al., 2015). Since temperature is an important factor for the adaptation and survival in the respiratory tract, as well as for the colonization properties and the virulence of *M. catarrhalis* (Heiniger et al., 2005; Spaniol et al., 2013), these results indicate that the levels of expression of this efflux pump are controlled by cues with relevance for the infectious success of *M. catarrhalis*. This further supports that efflux pumps may form part of global regulatory networks that include resistance and virulence determinants among other elements (Randall and Woodward, 2002; Luong et al., 2003; Nikaido et al., 2008; De Majumdar et al., 2013).

The ability to persist and to replicate in bile-rich environments is also critical for the pathogenesis of the food-borne pathogen *Listeria monocytogenes*. Among the elements that contribute to its survival in bile salts are the MDR efflux pumps MdrM and MdrT, belonging to the MFS family (Quillin et al., 2011). Expression of both MDR efflux pumps is strongly induced by cholic acid, a bile component, but only the MdrT efflux pump is able to extrude this compound, which is toxic for mutants lacking this efflux pump. Besides, MdrT is an important virulence factor involved in the colonization of the gallbladder *in vivo*, since mutants lacking *mdrT* are 100-fold attenuated. This study also suggests that MdrM has a synergistic role with MdrT in *L. monocytogenes* liver colonization, although MdrM substrates have not been identified yet (Quillin et al., 2011).

RND efflux systems also play a role in the pathogenesis of *Vibrio cholerae*. It has been reported that VexAB, VexCD, VexIJK, and VexGH contribute not only to antimicrobial resistance, but also to the colonization of the infant mouse small intestine; deletion of these systems impair the colonization of the mice intestine by *V. cholerae* (Bina et al., 2008; Taylor et al., 2012). Besides being relevant factors for colonization, these efflux pumps are required for the expression of the genes that encode two of the most important virulence factors in *V. cholerae*: the cholera toxin (CT) and the toxin-coregulated pilus (TCP). In a mutant lacking *vexB*, *vexD*, *vexH*, and *vexK*, the production of CT and TcpA (the pilin subunit of the TCP) is reduced by 45%, while a six RND-null strain showed a 70% reduction in the expression of these virulence determinants, suggesting that the remaining efflux pumps, VexF and VexM, also contribute to virulence in *V. cholerae* (Taylor et al., 2012).

Multidrug efflux systems are also relevant elements in the defense against oxidative stress produced in the host during phagocytosis. For example, the ABC family efflux pump MacAB is required for the survival of *S. enterica* serovar Typhimurium inside macrophages, where they are exposed to ROS. It has been observed that mutants lacking *macAB* showed an impaired intracellular replication in macrophages as compared with the wild-type parental strain and also failed in growing in the inflamed intestine, where neutrophils release ROS. Further, the same mutants were able to grow inside macrophages that do not produce ROS, which implies that MacAB is needed for *S. enterica* serovar Typhimurium replication inside macrophages and for the survival under oxidative stress conditions. Besides, the *macAB* deletion mutant had a defect in liver colonization in BALB/c mice after intraperitoneal infection, which indicates another function of this system in the infective program of *S. enterica* serovar Typhimurium (Bogomolnaya et al., 2013).

In addition to the role of efflux pumps in the interaction of bacterial pathogens with the compounds present in the host, they also may play a direct role in virulence. This is the case for the *Mycobacterium tuberculosis* RND proteins designated as MmpL (*M*ycobacterial *m*embrane *p*rotein *L*arge; Cole et al., 1998). The genome of *M. tuberculosis* encodes 13 of these proteins, which role seems to be transporting lipids for their incorporation on the cell envelope, providing protection against host-derived compounds and contributing to the bacterial virulence (Neyrolles and Guilhot, 2011). Domenech et al. (2005) examined the contribution of these proteins to the bacterium virulence by using a murine model of infection and mutants for each MmpL protein. Among these proteins, they found that MmpL4, MmpL7, MmpL8, and MmpL11 were required for the virulence maintenance, since there is an increase in the survival time when the host is infected with these mutants as compared with the wild-type strain. The attenuation of the *mmpL7* mutant might be due to the lack of phthiocerol dimycocerosate (PDIM), which is an abundant wax of the outer cell matrix involved in the cell permeability (Camacho et al., 2001); and the *mmpL8* mutant is deficient in SL-1 because MmpL8 is involved in the transport of SL-N, a precursor of SL-1 (Domenech et al., 2004). It has been shown that SL-N stimulates human CD1b-restricted T cells (Gilleron et al., 2004), a feature that might explain the attenuation of the *mmpL8* mutant if this molecule has a similar effect on murine CD1d-restricted T cells. The mechanisms by which *mmpL4* and *mmpL11* mutants were more attenuated have not been fully elucidated; however, it has been recently described a mutation in *mmpL4a* (Tyr842His) in *Mycobacterium bolletii*, which is responsible for the smooth-to-rough morphotype change, since MmpL4 is involved in the transport of glycopeptidolipid. This variant also contributes to the bacterial virulence in a zebrafish model (Bernut et al., 2016). The Tyr842 residue is conserved in all other mycobacterial MmpL4 orthologs and in all 13 MmpL RND proteins in *M. tuberculosis*, which indicates the functional relevance of this residue (Szekely and Cole, 2016).

Biofilms are complex microbial associations attached to a variety of surfaces. Bacteria that grow forming biofilms are more resistant to antibiotics than planktonic cells, being also important elements in the bacterial virulence and pathogenesis. The link between antimicrobial tolerance on biofilms and efflux pumps has been reported in several microorganisms (Soto, 2013). For instance, in the opportunistic pathogen *P. aeruginosa*, the MerR-like regulator BrlR plays a role in the high-level tolerance to antimicrobials in biofilms because it is able to activate under these growing conditions the expression of the multidrug efflux pumps MexAB-OprM and MexEF-OprN (Liao et al., 2013). A novel efflux pump in *P. aeruginosa* involved in biofilm tolerance, named as PA1874-1877, has been identified. The expression of this efflux pump was 10-fold increased in biofilms when compared with planktonic cells. Besides, deletion of the genes encoding this efflux pump resulted in an increased susceptibility to ciprofloxacin, gentamicin, and tobramycin (Zhang and Mah, 2008). Efflux pump expression can also impact the flagellar motility, which plays a relevant role in biofilm formation (Houry et al., 2012) and enhances pathogenicity by improving bacterial motility (Duan et al., 2013). In *Stenotrophomonas maltophilia*, another opportunistic pathogen, it has been observed that deletion of the RND efflux pump SmeYZ resulted in a reduced ability to form biofilm and the abolition of flagella formation. Besides, this deletion mutant was more susceptible to redox compounds, human serum and neutrophils, which indicates that this efflux pump is also involved in the protection against ROS (Lin et al., 2015).

Expression of efflux pumps not always enhance virulence; constitutive overexpression of these systems in antibiotic resistant mutants, can compromise the bacterial fitness and the virulence as well (Sanchez et al., 2002), indicating that the expression of these elements is finely regulated and deviations on this regulation, altering their expression below or above the physiological levels, may impair bacterial physiology and virulence. This is the case of the overexpression of the MDR efflux pumps MexCD-OprJ and MexEF-OprN in *P. aeruginosa*, which negatively affect the expression of the type III secretion system (T3SS; Linares et al., 2005). The T3SS is an important virulence mechanism, since bacteria are able to inject effector proteins manipulating the host cell function (Coburn et al., 2007). The effect of over-expression of efflux pumps on T3S was due to the lack of expression of *exsA* gene, which is the transcriptional activator of the T3SS in *P. aeruginosa* (Linares et al., 2005). More recently, it was found that MexT, the positive regulator of MexEF, was able to repress the expression of the T3SS through the regulators MexS and PtrC (Jin et al., 2011), in such a way linking resistance and virulence within a single regulon.

Altogether, these works show that, in addition of being involved in antibiotic resistance, efflux pumps can participate in bacterial virulence as well. In some cases, a direct effect can be foreseen; this is the situation of efflux pumps able to extrude host-produced antimicrobial compounds. However, in other occasions the reasons behind the effect of efflux pumps on virulence are not so straightforward. In some cases, the effect on virulence is derived from the integration of the efflux pumps in a regulon that also includes virulence determinants. This could be the case of MexEF, a part of the MexT regulon that also includes the *P. aeruginosa* T3SS. Mutations in this global regulator will simultaneously alter antibiotic resistance and virulence, although the efflux pump itself is not directly involved in T3S. Other situation, also described for MexEF, is the capability of some efflux pumps for extruding intercellular signal molecules (see above) or their precursors (Olivares et al., 2012). If this efflux pump is abnormally expressed, the levels of expression of the genes belonging to the regulatory network (frequently including virulence genes) triggered by such signals will be also altered.

## The Functional Role of Multidrug Efflux Pumps in Plant–Bacteria Interactions

**FIGURE 2:**
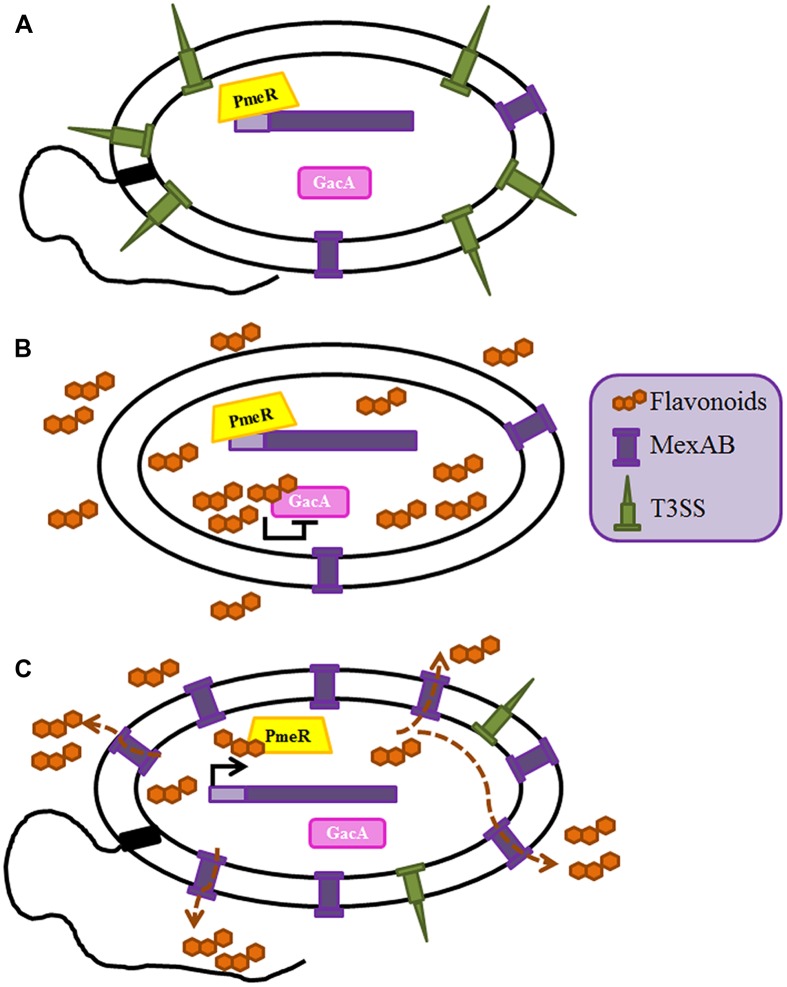
**Role of the MexAB-OprM efflux pump in the adaptive co-evolution of plant defense and *P. syringae* virulence.** The intracellular amount of the plant flavonoids is regulated by MexAB-OprM efflux pump. **(A)** Expression of *mexAB-oprM* is controlled by the transcriptional repressor PmeR; in the absence of flavonoids the expression of this efflux pump is repressed and expression of virulence determinants as the T3SS or bacterial motility is activated through the GacS/A TCS (Vargas et al., 2011). **(B)** The presence of flavonoids inhibits GacS/A, hence leading a transient low virulence phenotype, in which the expression of the T3SS and the motility are low (Vargas et al., 2013). **(C)** The accumulation of flavonoids inside the cell leads to over-expression of *mexAB-oprM* through the release of its transcriptional regulator PmeR from its operator. Since flavonoids are substrates of MexAB-OprM, under these conditions they are efficiently extruded and their intracellular concentration falls down below the threshold required for inactivating GacS/GacA. As a consequence of this situation, the virulent state of *P. syringae* is restored.

Multidrug efflux pumps, in addition of being relevant antibiotic resistance determinants, are relevant key players for the behavior of microorganisms in their natural (non-clinical) habitats. Indeed, whereas most studies in human pathogens have concentrated in the role on antibiotic resistance of these elements, the analyses of efflux pumps from plants pathogens or epiphytes, has mainly focused on their role in plant–bacteria or bacteria–bacteria interactions. The rhizosphere is a natural ecosystem that includes a complex microbiome formed by microorganisms that live in contact with plants’ roots. Roots’ and other plants’ exudates contain a large array of natural products, such as flavonoids, which confer them protection against microbial attack. However, different microorganisms have developed mechanisms to deal with the activity of those compounds. One of them is the flavonoid-responsive RND family of efflux pumps, which includes several members as MexAB-OprM from *Pseudomonas syringae*, AcrAB from *Erwinia amylovora*, AcrD from *Erwinia chrysanthemi*, IfeAB from *Agrobacterium tumefaciens*, XagID2689 from *Xanthomonas axonopodis*, SmeDEF from *S. maltophilia*, EmrAB from *Sinorhizobium meliloti* and BjG30 from *Bradyrhizobium japonicum* (Palumbo et al., 1998; Burse et al., 2004b; Vargas et al., 2011; Takeshima et al., 2013; García-León et al., 2014; Pletzer and Weingart, 2014; Rossbach et al., 2014; Chatnaparat et al., 2016). Some of these efflux pumps are implicated in plant colonization, whereas some others are involved in bacteria/plant symbiosis processes. As stated before MexAB-OprM is described as a significant determinant of multidrug resistance in *P. aeruginosa* (Poole, 2001) having a basal expression level enough to contribute to the intrinsic antimicrobial resistance of these bacteria (Li et al., 1995) and it also has a role in virulence. Indeed, as above described, mutant strains overexpressing this efflux pump are less virulent because of the extrusion of the QS homoserine lactone 3-oxo-C_12_-HSL (Minagawa et al., 2012). In addition of extruding antibiotics or QS signaling molecules, MexAB-OprM is able to pump out plant antimicrobial compounds from leaves of *Melaleuca alternifolia*, supporting that, in addition of its role in clinical settings, this efflux pump (and several others, see below) may allow bacterial survival in a vegetal environment (Papadopoulos et al., 2008). Indeed, this efflux pump is required for the efficient colonization of tomato plants by *P. syringae*, since the inactivation MexAB-OprM lead to a defective colonization capacity of the plant by this bacterial species (Vargas et al., 2011). In line with the role of efflux pumps in a general bacterial response to the plant antimicrobial defense, it has been shown that flavonoids are inducers and substrates of this transporter (Vargas et al., 2011). In addition, the same flavonoids are able to inhibit the GacS/GacA two component system (TCS) of *P. syringae*, which is implicated, among other regulatory elements, in the activation of the motility and the T3SS in this species (Chatterjee et al., 2003). In fact, the absence of MexAB-OprM and the accumulation of flavonoids inside bacteria lead to a reduction of swarming and swimming motility and a significant impairment in the production of flagella and T3S (Vargas et al., 2013). Therefore, when the amount of plant flavonoids is enough to reduce bacterial virulence by inhibiting GacS/GacA, *P. syringae* may extrude such flavonoids using the MexAB efflux pump (which expression is now induced), shrewdly regulating the intracellular level of flavonoids and consequently, ensuring the viability of the bacteria by the de-repression of this TCS. This is an elegant example of the adaptive co-evolution of plant resistance and pathogen virulence in which the role of this efflux pump goes beyond being a mere detoxification system (**Figure 2**).

Another plant-pathogenic bacteria is *E. amylovora*, an Enterobacteria that causes fire blight on apple and pear trees. The flavonoid inducible AcrAB-TolC efflux pump from *E. amylovora* confers resistance, in addition to antibiotics, to plant compounds as phytoalexins and flavonoids (phloretin, naringenin, and quercetin) leading to the successful colonization of plants (Burse et al., 2004b; Maggiorani Valecillos et al., 2006; Al-Karablieh et al., 2009). Moreover, an AcrAB deletion mutant has a strong reduction of *E. amylovora* virulence in apple plants (Burse et al., 2004b). A close homolog of AcrB is AcrD, an efflux pump that is also induced by plant flavonoids, such as luteolin, but whose deletion mutant exhibits full virulence on apple and pear fruits (Pletzer and Weingart, 2014). *E. chrysanthemi* constitutes another example in which efflux pumps are required to infect plants. A *tolC* mutant of *E. chrysanthemi* is unable to extrude the plant antimicrobial compound berberine, being then unable to cause plant tissue maceration. Moreover, TolC plays a role also modulating the fitness of the bacteria growing in the microbial community (Barabote et al., 2003), indicating that efflux pumps might also be involved in inter-microbial interactions (see below). In addition of plant-derived antimicrobials, the AcrAB efflux pump of *E. chrysanthemi* is inducible by salicylic acid (SA), an important plant hormone implicated in local and systemic plant resistance (Ravirala et al., 2007). SA and others plant phenolic acids are able of reducing the expression of the T3SS, by inhibiting the GacS/A pathway, in *P. syringae* (Lee J.S. et al., 2015), suggesting that a role of efflux pumps in non-clinical ecosystems might be to adjust the intracellular level of plant signals, as a mechanism to deal with plant defense systems. Other efflux pump that has a role in plant colonization is IfeAB from *A. tumefaciens*, which extrudes coumestrol bestowing measurable ecological benefits to this bacterium in flavonoids rich environments. *X. axonopodis* pv. *glycines* is another plant pathogen, causative of bacterial pustule of *Glycine max*, one of the most important diseases in soybean. It contains another flavonoid inducible RND efflux pump, called XagID2689 (Chatnaparat et al., 2016), which in addition of being induced by flavonoids contributes as well to flavonoids’ resistance; its deletion strongly reduces bacterial virulence in soybean. Moreover, this deletion mutant shows a higher susceptibility than the wild-type parental counterpart to the isoflavonoids phloretin, naringenin and berberine, as well as to the antibiotics acriflavine and tetracycline, suggesting a role in the intracellular reduction of the levels of several compounds produced by soybean and involved in its antimicrobial response program. In *S. maltophilia*, the SmeDEF efflux pump, which is the most important quinolone resistance determinant of this microorganism (Alonso and Martinez, 2000, 2001; Zhang et al., 2001; Garcia-Leon et al., 2014), is induced by flavonoids, that are able to bind to its SmeT repressor (García-León et al., 2014); in addition, a mutant lacking *smeE* is unable to colonize the roots of *Arabidopsis* plants (García-León et al., 2014), further supporting that MDR efflux pumps, with a relevant role for antibiotic resistance at clinical settings, might have been selected in nature for different ecological purposes.

In addition of easening the bacterial plants’ colonization and infection, efflux pumps have also a role in the interactions between plants and their symbiotic bacteria. For example, EmrAB from *S. meliloti* is an inducible flavonoid efflux pump with a role in symbiosis with *Medicago sativa*; the symbiotic process is impaired when the regulator of this efflux system, the TetR repressor EmrR, is deleted (Rossbach et al., 2014; Santos et al., 2014). Other examples of efflux pumps with a role in symbiotic nitrogen-fixation activity processes in *G. max* are BdeAB and BjG30 from *B. japonicum* (Lindemann et al., 2010; Takeshima et al., 2013). BdeAB deficient mutants, in addition of presenting symbiotic defects, are more susceptible to aminoglycosides, highlighting the multifunctional role of efflux pumps (Lindemann et al., 2010).

Efflux pumps have also an important role in the inter-microbial interactions in the host plant and in its rhizosphere, where each bacteria have to compete for space and nutrients to survive. As with antibiotic resistance mechanisms, that can be considered as a colonization factor in the treated patient, where antibiotics are present (Martinez and Baquero, 2002), resistance to antimicrobials produced by epiphytes may be considered as a colonization tool of phytopathogenic bacteria and *vice versa*. If the epiphytes can inhibit the phytopathogens, the plant will be protected from infection, whereas if pathogens can inhibit epiphytes, this situation will provide a colonization advantage and hence better possibilities for infecting the plant. Resistance to compounds produced by bacterial competitors will then increase the chances for colonization of the epiphyte or the pathogen, with important consequence in terms of crops protection. This is the case of the efflux pump NorM from *E. amylovora* that, in contrast to the previously mentioned efflux pump AcrAB from *E. amylovora* (Burse et al., 2004b), is not able to extrude the high amount of isoflavonoids that produce the members of the Rosaceae family (Burse et al., 2004a). Moreover, a *norM*-deficient mutant causes comparable symptoms as the wild-type parental counterpart in plant tissues, indicating that this efflux pump does not contribute to virulence of *E. amylovora* against apple plants. Nevertheless, while the NorM efflux pump does not directly contribute to virulence *E. amylovora*, it has an important role extruding toxic molecules produced by *Pantoea agglomerans* (Pusey et al., 2011), an epiphytic bacteria that is an excellent colonizer of stigmas of apple and pear and it may effectively inhibit the multiplication of *E. amylovora* when both microorganisms co-colonize rosaceous (Burse et al., 2004a; Piddock, 2006b), hence constituting a potential biocontrol agent for fire blight (Kim et al., 2012). It is worth mentioning that NorM is involved in the capability *E. amylovora* to reach high-density populations at low temperatures. Indeed, at 18°C the growth of an *E. amylovora norM* mutant is significantly impaired as compared with the wild-type strain. In addition, the level of expression of *norM* is twofold greater at 18°C gene than at 28°C (Burse et al., 2004a). Altogether these results suggest that the intrinsic resistance of *E. amylovora* to *P. agglomerans* competition at 18°C may be due to the increased expression level of *norM*, which might allow the colonization by *E. amylovora* of the stigma surface of the blossom when it coexists with *P. agglomerans*. These evidences lead to hypothesize that the utilization of *P. agglomerans* as a biocontrol mechanism for fire blight would be compromised at low temperatures or by mutations causing of overexpression of *norM* in *E. amylovora*.

## Author Contributions

All authors listed, have made substantial, direct and intellectual contribution to the work, and approved it for publication.

## Conflict of Interest Statement

The authors declare that the research was conducted in the absence of any commercial or financial relationships that could be construed as a potential conflict of interest.
